# Table Olives Fermented in Iodized Sea Salt Brines: Nutraceutical/Sensory Properties and Microbial Biodiversity

**DOI:** 10.3390/foods9030301

**Published:** 2020-03-06

**Authors:** Barbara Lanza, Sara Di Marco, Nicola Simone, Carlo Di Marco, Francesco Gabriele

**Affiliations:** 1 Council for Agricultural Research and Economics (CREA), Research Centre for Engineering and Agro-Food Processing (CREA-IT), Via Nazionale 38, 65012 Cepagatti (PE), Italy; sarettadimarco87@gmail.com (S.D.M.); nicola.simone@crea.gov.it (N.S.); carlo.dimarco@crea.gov.it (C.D.M.); 2 Azienda Agricola Francesco Gabriele, Via Praino Agostino 1, 87076 Villapiana (CS), Italy; info@francesco-gabriele.com

**Keywords:** table olives, minerals, sea salt, PGI, iodized salt, functional food

## Abstract

This research aimed to study the influence of different brining processes with iodized and noniodized salt on mineral content, microbial biodiversity, sensory evaluation and color change of natural fermented table olives. Fresh olives of *Olea europaea* Carolea and Leucocarpa cvs. were immersed in different brines prepared with two different types of salt: the PGI “Sale marino di Trapani”, a typical sea salt, well known for its taste and specific microelement content, and the same salt enriched with 0.006% of KIO_3_. PGI sea salt significantly enriches the olive flesh in macroelements as Na, K and Mg, and microelements such as Fe, Mn, Cu and Zn. Instead, Ca decreases, P remains constant, while iodine is present in trace amounts. In the olives fermented in iodized-PGI sea salt brine, the iodine content reached values of 109 μg/100 g (Carolea cv.) and 38 μg/100 g (Leucocarpa cv.). The relationships between the two varieties and the mineral composition were explained by principal component analysis (PCA) and cluster analysis (CA). Furthermore, analyzing the fermenting brines, iodine significantly reduces the microbial load, represented only by yeasts, both in Carolea cv. and in Leucocarpa cv. Candida is the most representative *genus*. The sensory and color properties weren’t significantly influenced by iodized brining. Only Carolea cv. showed significative difference for b* parameter and, consequently, for C value. Knowledge of the effects of iodized and noniodized brining on table olives will be useful for developing new functional foods, positively influencing the composition of food products.

## 1. Introduction

Table olives are a typical food product in the “Mediterranean diet”, edible as finger food directly or as an ingredient for more complex dishes. In Italy, during the last five years, the average consumption of table olives was approximately 115,000 tons/year with a per capita consumption of 1.9 kg/year. Italian production covers only 50.9% of consumer demand; the remaining part is imported from Spain, Greece and Tunisia. Italy is rich in typical table olive products [1], obtained by traditional methods, and many of those have obtained or are likely to obtain to the recognition of the PDO (Protected Designation of Origin) or PGI (Protected Geographical Indication) trademarks. Currently, four Italian PDO are recognized: “Nocellara del Belice” (Reg. EC 134/1998), “La Bella della Daunia” (Reg. EC 1904/2000), “Oliva Ascolana del Piceno” (Reg. EU 1855/2005) and “Oliva di Gaeta” (Reg. EU 2016/2252). The processing of table olives also has long been part of Mediterranean traditional food and food industry. Table olives can be produced using different processes that vary according to many parameters; the most applied processes in Italy are the “Sevillan-style” and “Castelvetrano-style” for green treated olives and the “Greek-style” for green, turning color and black olives [2]. Referring to the new tendencies in “functional foods” that are intended to produce foods that contain value-added compounds as vitamins, microelements and other healthy substances (i.e., radical scavenging molecules), it appears to be quite clear that table olives already have most of the characteristics required to properly join the group due to their composition, i.e., high bio-phenol content with antioxidant and radical scavenging activity, vitamins, MUFA, PUFA, minerals and other nutraceutical compounds. Table olives contain simple and complex phenolic compounds (at least 30 different phenolic compounds) in amounts ranging between 100 and 350 mg/100 g of e.p. (edible portion). This quantity is the same of 1 kg of extra virgin olive oil. The polyphenol content and composition depend on several factors such as cultivar, stage of ripening, location and processing [1,3]. The ratios MUFA/SFA (3.3–6.8), PUFA/SFA (0.2–0.8), cis-MUFA + cis-PUFA/SFA + TFA (3.5–7.3), oleic acid/palmitic acid (3.6–8.0) and ω6/ω 3 (6.7–23.5) are used to assess the nutritional quality of the lipid fraction in foods having a regulatory influence on certain thrombogenic and fibrinolytic markers during the postprandial state in healthy subjects [1]. 

Iodine is an important micronutrient element and is required for the synthesis of T4 and T3 thyroid hormones. An iodine-deficient diet causes a wide spectrum of illnesses, including goiter and mental retardation. Adolescents and adults need iodine in amounts of 150 μg/day (Table 1). The oral intake also includes iodine from water and beverages; however, food provides by far the most to the total iodine absorption. The iodine contents in the principal categories of foods are summarized in Table 2. The iodine content in sea fishes and seafood is high, reflecting the content in the water they inhabit [4]. 

Iodine content in vegetable foods is lower compared to those of animal origin due to a low iodine concentration in soil. As consequence, 80% of the vegans suffer from iodine deficiency [9].

Iodized salt has been used in food processing to prevent iodine deficiency disorders. In Italy, current legislation requires the iodization of salt for direct human consumption or as an ingredient in the preparation and storage of food products (Law n.55 of 21 March 2005). Moreover, table olives are mostly fermented, so they contain a good quantity of yeasts and bacteria involved in the fermentation processes [10,11,12] that could have a probiotic activity on human organisms [13,14,15]. Little information is available on the effects of iodine in the fermentation process and associated microbiota [16].

In this work, we focused our research on a typical production, consisting of natural processing fermented table olives obtained by two different cultivars, i.e., cv. Leucocarpa (white olives) and cv. Carolea, using two different types of salt for brine preparation, i.e., the PGI “Sale marino di Trapani”, a typical sea salt, well known for its taste and specific content in microelements, and the same salt enriched with 0.006% of KIO_3_. The olive samples were analyzed by looking for differences in chemical composition with regard to macro–micro element enrichment, in particular iodine content, to develop a new functional food which is well characterized from the sensory point of view. We also investigated the microbiological composition of the different fermentation brines, looking for differences in the microbial pools involved in the fermentation process.

## 2. Material and Methods

### 2.1. Samples and Treatments

Fresh olives of *Olea europaea* “Carolea” and “Leucocarpa” cvs. were harvested in December at full ripening (Carolea fruits were purple-black while Leucocarpa fruits were ivory-white). Two samples for each cultivar were submerged in two different 8% brines prepared with (a) PGI “Sale marino di Trapani” sea salt; (b) the same sea salt enriched with 0.006% of KIO_3_ (corresponding to 3.7 mg of iodine/100 g of salt). The Protected Geographical Indication (PGI) “Sale marino di Trapani” (Reg. EU 1175/2012) is a sea salt obtained through the fractional precipitation of the compounds contained in seawater by evaporation, within the salt pans of Trapani (Sicily, Italy), without additives, bleaches, preservatives or anticaking agents. It is very rich in mineral macro and microelements (Table 3). 

At the end of fermentation (after 8 months), representative samples were analyzed for the mineral content, microbial biodiversity, sensory attributes and color change.

### 2.2. Mineral Composition

Representative samples were analyzed in order to quantify the minerals content, according to the procedures described by Lopez, Garcia and Garrido [17]. Iodine levels were determined according to a method described by Amr and Jabay [18]. The analyses were carried out in duplicate and the results expressed as mg/kg of olive fresh pulp. 

### 2.3. Microbiological Monitoring

To study the microbial diversity (total microflora, yeasts, molds, and lactic acid bacteria), serial dilutions with distilled water were prepared from each brine and plated on agar media. Total microflora was grown on Plate Count Agar (PCA; Oxoid, Basingstoke, UK) incubating the plates at 30 °C for 72 h; yeasts on Malt Extract Agar (MEA; Oxoid) at 30 °C for 48 h; lactic acid bacteria on Man, De Rogosa and Sharpe (MRS; Oxoid) at 30 °C for 48 h in anaerobic atmosphere. Culture responses were expressed as colonies forming units (CFU) per ml of brine. 

At least five yeast colonies from each brine were isolated and subcultured in MEA. Biochemical identification was carried out by API 20 C AUX (bioMerieux SA, Marcy-l’Etoile, France) and, in case of uncertain classification, by RapID Yeast Plus System (Remel, Lenexa, KS, USA).

The API 20 C AUX system consists of 20 cupules containing dehydrated substrates which enabled us to perform 19 assimilation tests. After 72 h of incubation, in case of positivity, inoculum suspensions generate turbidity changes. The API on-line database (api*web*^TM^) was used for species identification and an associated probability was assigned to each culture.

RapID Yeast Plus system uses a qualitative micromethod with 18 conventional and chromogenic substrates. Based on chromogenic changes, a microcode, which was entered into the Remel database (ERIC^TM^) for species identification with an associated probability for each culture.

### 2.4. Sensory Evaluation of Table Olives

The sensory characteristics of table olives were evaluated by tasters of the CREA-IT Panel, according to the COI/OT/MO No 1/Rev. 2 [19]. The evaluated attributes were negative sensations (abnormal fermentation, cooking effect, rancid, musty, or other defects), gustatory sensations (salty, bitter, sour) and kinesthetic sensations (hardness, fibrousness, and crunchiness). The table olive profile sheet uses a 10 cm intensity scale ranging from 1 (no perception) to 11 (extreme). 

### 2.5. Determination of Color

The surface color of the fruits was measured using a Color-view spectrophotometer (Konica Minolta Optics, 2970 Ishikawa-machi, Hachioji, Tokyo, Japan; Model CM-2600D). Color was expressed in terms of CIE (Commission Internationale de l’Eclairage) L* (lightness), a* (redness/greenness), b* (yellowness/blueness) and their derivative Chroma (C = √a*^2^ + b*^2^). The analysis of color was made on 20 uniformly sized olive fruits. 

### 2.6. Statistical Analyses

All data significance was evaluated by one-way ANOVA using the F-test (*P* ≤ 0.05).

Mineral data are processed by principal component (PCA) and cluster (CA) analyses, carried out in the Past PAleontological STatistics software (Version 2.12, Øyvind Hammer, Natural History Museum, University of Oslo). For data preprocessing, the variables were rescaled from 0 to 1. 

In order to elaborate the sensory data, a method was applied to calculate the median (Me), the robust standard deviation (DSr), the robust coefficient of variation percentage (CVr%), and the confidence intervals of the median at 95% (C.I._upper_ and C.I._lower_) contained in Annex 1 [19], taking into account those attributes with a robust coefficient of variation of 20% or less. 

## 3. Results and Discussion

**Figure 1:**
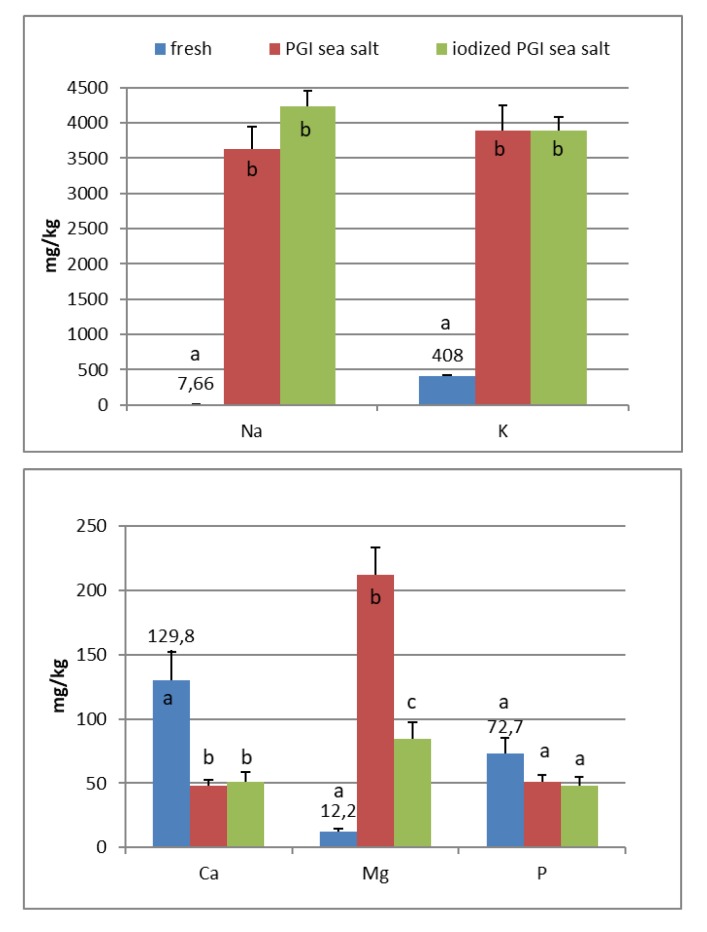

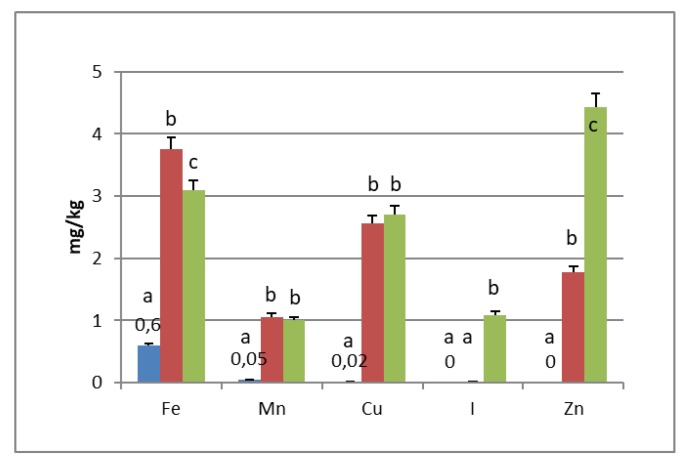
Mineral composition of fresh and processed Carolea olives. Data are expressed in mg/kg. Bars represent mean values of two replicates ± SD. Significant differences are indicated by different letters (*P* < 0.05).

**Figure 2:**
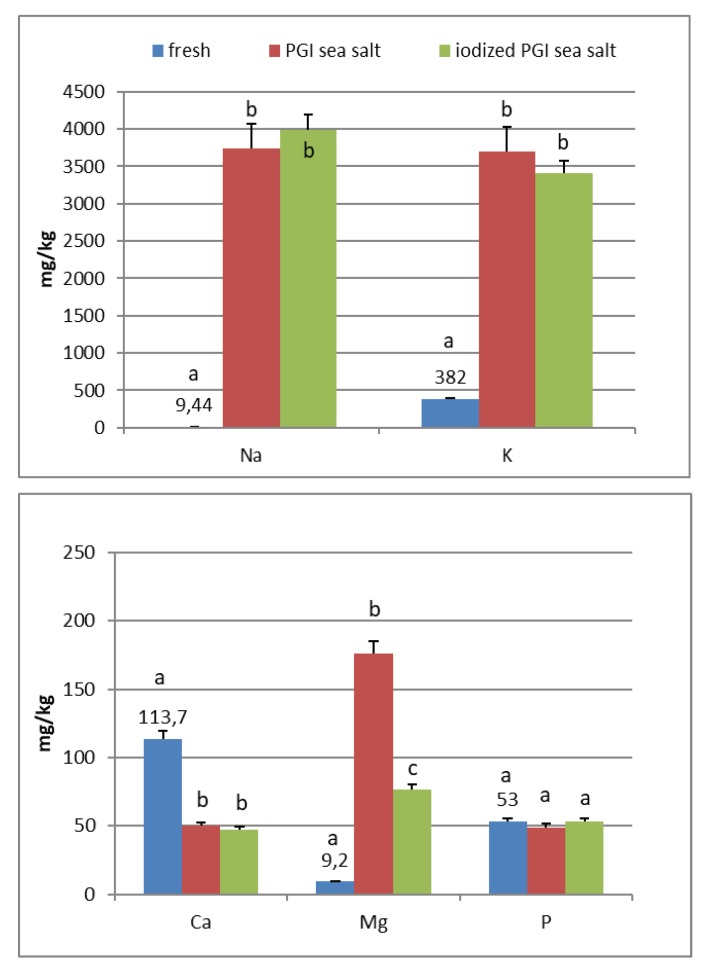

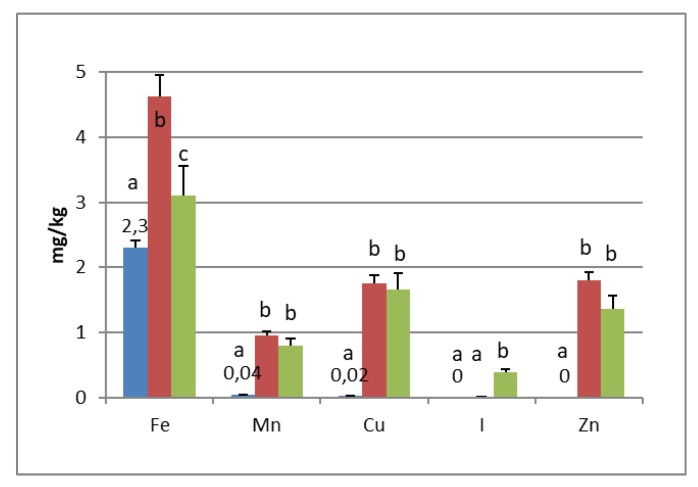
Mineral composition of fresh and processed Leucocarpa olives. Data are expressed in mg/kg. Bars represent mean values of two replicates ± SD. Significant differences are indicated by different letters (*P* < 0.05).

In the fresh fruits of Carolea cv. (Figure 1), the main composition (expressed in mg/kg) was: K (408), Ca (130), P (73), Mg (12), and Na (8). Fe, Mn, Cu, Zn, and I are <1. In the fresh fruits of Leucocarpa cv. (Figure 2), the main composition (expressed in mg/kg) is: K (382), Ca (114), P (53), Na (9), Mg (9), and Fe (2). Mn, Cu, Zn, and I are <1. Variation in mineral levels of fresh fruits depends of olive variety, ripening, and growing conditions (soil, water, fertilizers). With the addition of sea salts, minerals infuse into the olive flesh. The results of analysis indicated that the Na, K, Mg, Fe, Mn, Cu, and Zn contents increased during brining.

At the end of fermentation (Figure 1 and Figure 2), PGI sea salt brine principally enriched the olive flesh of macroelements Na, K, and Mg, reaching values of 3624, 3897, and 212 mg/kg (Carolea cv.) and 3733, 3696, and 176 mg/kg (Leucocarpa cv.) respectively.

The sodium content (<450 mg/100 g of edible portion) was shown to be below the allowances recommended by Italian Society of Human Nutrition [5], European Food Safety Authority [6], and the European Parliament and the Council of the European Union [8] (Table 1). The consumption of table olives would not be recommended only in cases of hypertension, and, in any case, there are production technologies to reduce salt content (low-sodium olives) [20,21,22]. As reported by other authors, the potassium concentration is higher in directly brined olives (571 to 1176 mg/kg) [17], which are not subjected to lye treatments. Values reported by De Castro Ramos et al. [23] for green olives ranged from 640 to 1090 mg/kg. Unal and Nergiz [24] found 3760 mg/kg for natural black olives. Biricik and Basoglu [25] reported a concentration of 4123–7401 mg/kg for green olive brine. The K content of different Turkish table olives varied between 2814 and 3386 mg/kg [26]. Despite daily intakes for K being high (2000–3500 mg) (Table 2), our fermented olives may be considered a main source for the daily allowance. From literature, magnesium concentration ranged from 51 to 197 mg/kg [17]. Its wide interval of concentration reflects that its presence may be greatly affected by processing. De Castro Ramos et al. [23] found 60–400 mg/kg in green olives and 47–360 mg/kg in Biricik and Basoglu [25]. The Mg content of different Turkish table olives varied between 83 and 156 mg/kg [26]. Sahan et al. [27] found values for black olives that range between 36 and 125 mg/kg.

PGI sea salt also significantly enriches the olive flesh of the microelements Fe (3.8 and 4.6 mg/kg for Carolea and Leucocarpa respectively), Mn (1.06 and 0.95), Cu (2.56 and 1.76), and Zn (1.78 and 1.80). Instead, Ca significantly decrease (about 50 mg/kg for cultivars), P content remains practically constant, while I content was not detectable (Figure 1 and Figure 2). The decrease in calcium can be explained by the subtraction of calcium ions from the olive flesh by Na+ and the formation of calcium salts (mainly CaCl2) in brine [28]. 

Iron concentrations in natural table olives are relatively low. Values reported by other authors were 3.49–7.70 [17], 6.4–10.9 mg/kg (green olives) [24], and 3.23–15.10 mg/kg for Turkish cultivars [26,27]. Manganese contents were always low in the same order as those given by other authors, i.e., 0.24–1.10 mg/kg [17], 1.40–2.72 [26], and 0.70–2.90 mg/kg for black table olives [29]. Copper concentrations in directly brined olives ranged between 3.99 and 10.93 mg/kg [17], 0.53 and 7.19 mg/kg for Turkish olives [26,27], and 7.00 and 30.00 mg/kg for black olives [29]. Zinc concentrations were like iron and copper in the same order as those given by other authors: 2.18–4.10 mg/kg [26], 4.25–14.30 [27], 1.55–3.20 [17], and 1.00–6.80 for black table olives [29]. Differences among cultivars within elaboration types were found. Analyzing the data present in the bibliography, phosphorus had a concentration that ranged from 57 to 144 mg/kg. Its highest average concentration was found in directly brined olives [17]. The P content varied between 116 and 250 mg/kg in different Turkish table olives [27]. The content of Ca in table olives ranged from 337 to 850 mg/kg [17], 422 to 850 mg/kg in green Turkish olives [26], 460 to 860 mg/kg in the green Spanish cultivar [23], 270 to 450 mg/kg for Kalamata, or 110 to 230 mg/kg for natural black olives after fermentation [24]. We haven’t found data on iodine content in either fresh and processed olives. 

In the olives fermented in iodized PGI sea salt, the iodine content reached values of 109 μg/100 g (Carolea cv.) and 38 μg/100 g (Leucocarpa cv.). The iodine retention in olive flesh contributes to meeting the recommended daily level for I (Table 1). Similar values were found in pickled vegetables (carrots, cucumbers, turnips, and cauliflowers). In this case, the iodine content ranged from 1.6 to 1.8 mg/kg [18]. The addition of KIO_3_ to the salt used for brining affects the redistribution of macro and microelements and, in particular, of magnesium, iron and zinc. The magnesium and iron contents significantly decreased (Figure 1 and Figure 2); this decrease in the olive flesh can be explained by the formation of Fe(IO_3_)_3_ and Mg(IO_3_)_2_ tetrahydrate or decahydrate. The concentrations of the various elements in the flesh depend on a favorable Ksp (solubility product constant) of the different salts that can be formed. In contrast, the zinc content significantly increased in Carolea cv. (Figure 1), but not in Leucocarpa cv. (Figure 2). However, the attained values are in the same order as those given by other authors [24,28]. The simultaneous presence of several counterions determined the final concentrations of the different elements in the flesh, depending on the relative concentrations of IO_3_^−^, K^+^, Mg^++^, Fe^+++^, and Zn^++^. 

The relationships between the two varieties and the three different processing steps were shown by principal component analysis (PCA) and cluster analysis (CA). 

**Figure 3:**
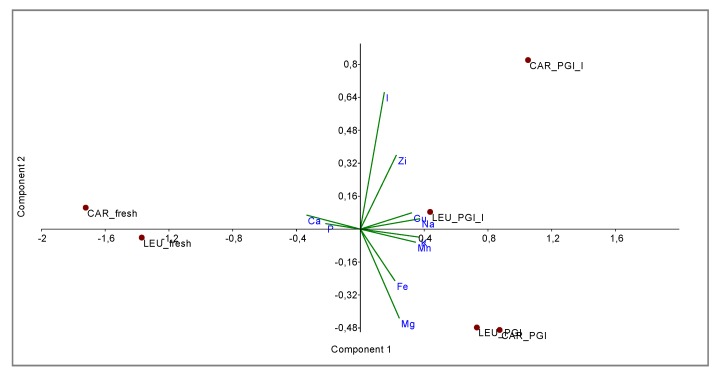
*Bi*-plot obtained by PCA of data-set based on mineral composition. CAR_fresh: Carolea fresh, LEU_fresh: Leucocarpa fresh; CAR_PGI: Carolea fermented in PGI sea salt; LEU_PGI: Leucocarpa fermented in PGI sea salt; CAR_PGI_I: Carolea fermented in PGI iodized sea salt; LEU_PGI_I: Leucocarpa fermented in PGI iodized sea salt.

**Figure 4:**
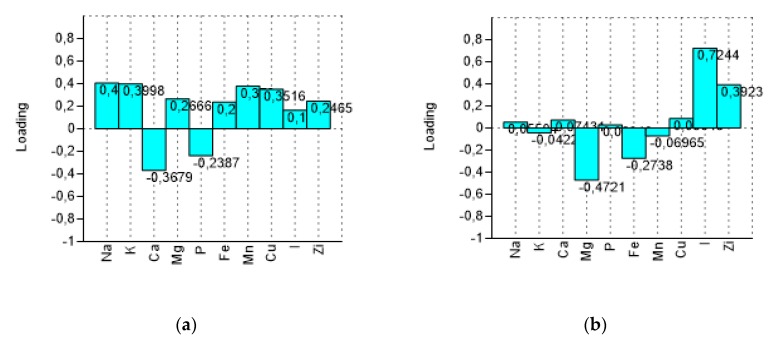
Loadings of variables (minerals) on the first two components (PC1 and PC2). (**a**) loading plot on PC1 and (**b**) loading plot on PC2.

PCA *bi*-plot (Figure 3) shows a good separation in relation to the processing steps for the variables (minerals). The variance was determined to be 93.40% by summing PC1 and PC2, reaching a value of 97.68% with PC3. In Figure 4 the influence of the variables on the construction of PC1 and PC2 is represented. With PC1, the higher variance contribution (about 0.40) was due to Na, K, Mn, Cu, and Ca. The latter is negatively correlated with other descriptors. With PC2, the major modules belonged to I (>0.70), Mg (−0.47), and Zi (0.39). I and Zn contribute to segregate olives processed with iodized brines. Magnesium is negatively correlated to the PC2. 

**Figure 5:**
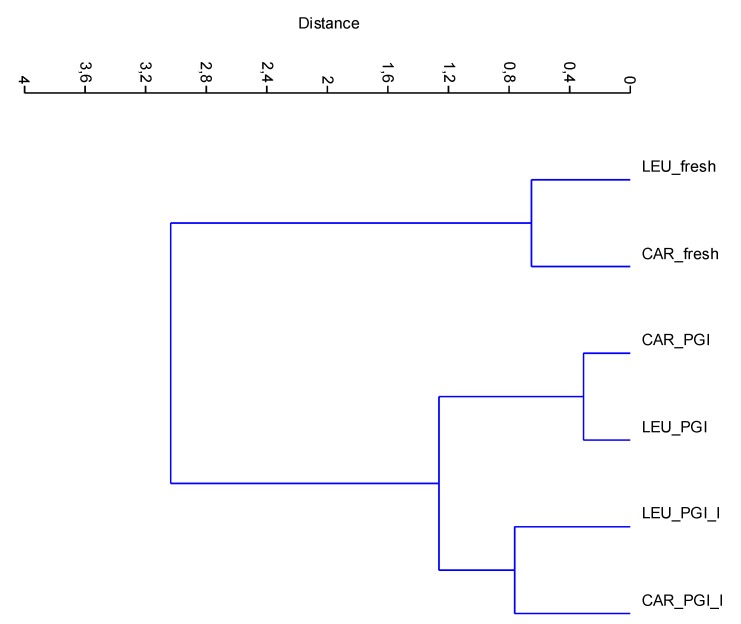
CA dendrogram of data-set based on mineral composition. Dendrogram is obtained using the Ward’s algorithm and the Euclidean distance similarity. CAR_fresh: Carolea fresh, LEU_fresh: Leucocarpa fresh; CAR_PGI: Carolea fermented in PGI sea salt; LEU_PGI: Leucocarpa fermented in PGI sea salt; CAR_PGI_I: Carolea fermented in iodized PGI sea salt; LEU_PGI_I: Leucocarpa fermented in iodized PGI sea salt.

The new data matrix of PCs was subjected to Cluster Analysis. Using the Ward’s method of clustering, the samples were grouped in three clusters (calculated at 1.0 of similarity distance) related to the processing steps. The obtained dendrogram is shown in Figure 5. The samples fermented in the iodized brines (CAR_PGI_I and LEU_PGI_I) were close to those fermented in simple sea salt, but were grouped into a separate cluster; they were very different from the fresh samples. This indicates considerable similarities between treatments but not between cultivars, i.e., the cultivar is a nondiscriminant attribute.

From the literature, there is no evidence that the use of iodized salt in processed foods production, including olives, could cause adverse changes in color and kinesthetic properties [18,30,31]. 

From our color data, Leucocarpa olives processed with PGI sea salt or with iodized PGI sea salt showed significative differences for all color parameters established by CIE (Table 4). In contrast, Carolea cv. showed significant differences only for b* and, consequently, for C values between PGI sea salt and iodized PGI sea salt, respectively (Table 4). 

**Figure 6:**
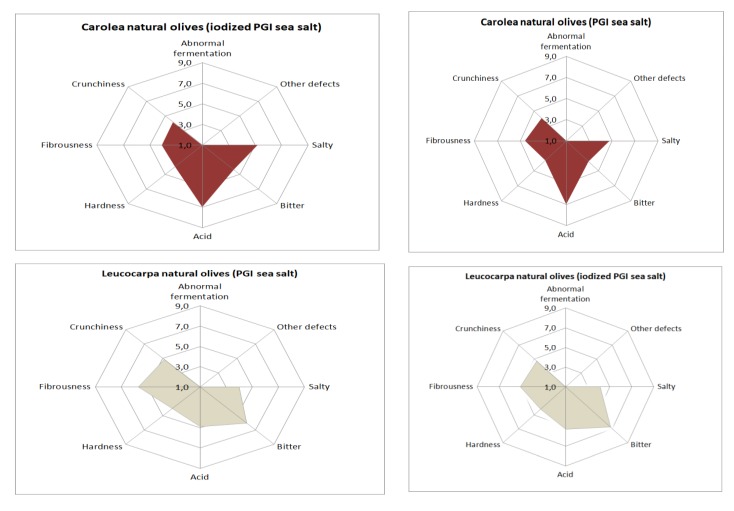
Sensory profiles of processed table olives.

Concerning sensory aspects (Figure 6), none of samples presented any defects. This is very important because the occurrence of negative sensations has a negative impact on the gustative and kinesthetic attributes [32]. Olives prepared with iodized sea salt were harder and more bitter than those prepared with noniodized sea salt, but these differences were not significant (Table 5). The bitterness level of Leucocarpa cv. is high, depending on the variety, but some groups of consumers prefer natural olives with high bitterness values. The low level of hardness highlighted by a sensory evaluation could be related to olive softening. From the data in our possession, there is no evidence that this level is problematic for commercialization.

A microbiological analysis of the fermented brines showed that lactobacilli and aerobic bacteria were absent; the total microflora consisted exclusively of yeasts. Iodized brines significantly reduce the microbial load both in Carolea cv. (8.7 × 10^4^ vs. 3.5 × 10^5^ CFU/mL) and in Leucocarpa cv. (1.4 × 10^2^ vs. 3.1 × 10^2^ CFU/mL) (Table 6). 

At the end of fermentation, *Candida* was the most representative genus, followed only by the genus *Cryptococcus*. As indicated in Table 7, the genus *Candida* was present in all four samples. In the Carolea cultivar only, *Candida krusei* was present (Table 7). The Leucocarpa cultivar showed a greater diversity in yeasts (*Candida famata*, *C. boidinii*, *C. intermedia*, *C. krusei,* and *Cryptococcus albidus*) (Table 7), indicating that these microorganisms are more related to the cultivar than to the environment. These yeasts have pectinolytic and cellulolytic enzymes and contribute to the degradation of the pectin that forms the middle lamella, which leads to cell separation and acts on cellulose, hemicellulose, and polysaccharides, giving texture to the pulp [33]. The low Ca content in processed olives (Figure 1 and Figure 2) could be related to the loss of Ca^2+^ bridging between residues of galacturonic acid of adjacent pectic chains, which, in turn, is related to the softening of the olive fruit [34].

*C. boidinii* contributes to the decline of olive tissue structural integrity [35] through its pectinolytic enzymes that act on pectic substances that form the middle lamella, and on cellulose, hemicellulose, and polysaccharides, that form the cell walls [36].

Each species of *Candida* requires a carbon source from different carbohydrates to provide energy for cell growth. The need of different carbohydrates sources becomes the basic identification for the assimilation method by every *Candida* species [37].

*C. krusei* identified by API 20 C AUX, deriving from Carolea brine samples, showed a positive reaction to glucose, *p*-Nitrophenyl phosphate, proline β-naphthylamide, and histidine β-naphthylamide. Sample 3 of Leucocarpa (iodized sea salt) brine had the same profile. *Cryptococcus albidus* and *C. famata* from Leucocarpa brine, identified only by RapID, grew on glucose, threalose, *p*-nitrophenyl-β,d-glucoside, *p*-nitrophenyl phosphate. *Cr. albidus* shows a positive reaction to *p*-nitrophenyl-β,d-fucoside (not for *C. famata*), and *C. famata* uses proline β-naphthylamide (not for *Cr. albidus*). Sample 5 (PGI sea salt) and samples 2 and 5 (iodized PGI sea salt) of Leucocarpa were identified as *C. intermedia* by RapID. These yeasts grow on glucose, glycerol, threalose, d-cellobiose, l-arabinose, adonitol, d-sorbitol, *N*-acetyl-Glucosamine, *p*-nitrophenyl-β,d-glucoside, *p*-nitrophenyl phosphate, proline β-naphthylamide, and histidine β-naphthylamide. Four cultures from Leucocarpa brine were identified by API as *C. boidinii*. These yeasts species showed a positive reaction to glucose, glycerol, d-xylose, adonitol, xylitol, d-sorbitol, and ρ-nitrophenyl phosphate. Furthermore, sample 4 of Leucocarpa (PGI sea salt) used histidine β-naphthylamide; samples 1 and 4 of Leucocarpa (iodized sea salt) used proline β-naphthylamide and histidine β-naphthylamide.

## 4. Conclusions

This research aimed to study the influence of different brining processes on mineral content, microbial biodiversity, sensory evaluation, and color change of natural fermented table olives. Fresh olives of *Olea europaea* Carolea and Leucocarpa cvs. were submerged in two different 8% brines prepared with iodized and noniodized PGI sea salts. Noniodized sea salt brines principally enriched the olive flesh with macroelements such as Na, K, and Mg, and microelements as Fe, Mn, Cu, and Zn. In contrast, a decrease in Ca was observed, while the P content remained practically constant, and I was present in trace amounts. In the olives fermented in iodized PGI sea salt, the iodine content reached the values of 109 μg/100 g (Carolea cv.) and 38 μg/100 g (Leucocarpa cv.).

The addition of KIO_3_ to the salt used for brining affects the redistribution of macro and microelements and, in particular, of magnesium, iron, and zinc. The concentrations of the various elements in the flesh depend on the favorable Ksp (solubility product constant) of the different salts that can form. 

The PGI sea salt enriches the olive fruit flesh with numerous microelements compared to simple brine prepared with only NaCl (data not shown). Many Italian companies utilize PGI sea salt only, instead of simple NaCl. The aim of our study was to determine whether the addition of KIO_3_ transfers iodine to the olive flesh.

Analyzing the fermenting brines, iodine significantly reduces the microbial load, represented only by yeasts, both in Carolea cv. and in Leucocarpa cv. *Candida* is the most representative genus. The sensory and color properties were not significantly influenced by iodized brining. Only Carolea cv. showed significant differences for the b* parameter and, consequently, for the C value.

This explorative research involved a small number of olive samples, but it compels us to undertake further investigations concerning the side effects of different salts on the microorganisms involved in the fermentation process, and the development of new functional foods that merge tradition and innovation. The results obtained represent the first data on the enrichment of iodine in table olives. 

## Figures and Tables

**Table 1:** Intakes and allowances recommended by SINU [5], EFSA [6] and EU [7].

Mineral	SINU-LARN ^1^	EFSA-DRVs ^2^	EU-RDA ^3^
NaCl (g/day)			6
Sodium (g/day)	1.2–1.5 (AI ^4^)	1.5 (AI)	
Potassium (mg/day)	3900 (AI)	3500 (AI)	2000
Calcium (mg/day)	1000–1200 (PRI ^5^)	950 (PRI)	800
Magnesium (mg/day)	240 (PRI)	350 (AI)	375
Phosphorus (mg/day)	700 (PRI)	550 (AI)	700
Iron (mg/day)	10 (PRI)	11–16 (PRI)	14
Manganese (mg/day)	2.3–2.7 (AI)	3 (AI)	2
Copper (mg/day)	0.9 (PRI)	1.3–1.6 (AI)	1
Iodine (μg/day)	150 (AI)	150 (AI)	150
Zinc (mg/day)	9–12 (PRI)	7.5–16.3 (PRI)	10

^1^ LARN: Italian reference intake levels for nutrients; ^2^ DRVs: Dietary Reference Values for nutrients; ^3^ RDA: Recommended Daily Allowance; ^4^ AI: Adequate Intake; ^5^ PRI: Population Reference Intakes.

**Table 2:** Iodine content in foods by FAO/WHO [8].

Food	FAO/WHO (µg/g)
Fish (marine)	163–3180
Fish (fresh water)	17–40
Shellfish	308–1300
Eggs	93
Milk	35–56
Meat	27–97
Cereal grains	22–72
Legumes	23–36
Vegetables	12–201
Fruits	10–29

**Table 3:** Composition of PGI “Sale Marino di Trapani” sea salt.

Composition	Units of Measurement	PGI “Sale Marino di Trapani” Sea Salt	Limits (Reg. EU 1175/2012)
Insoluble residue	%	0.07	<0.2
Residual moisture	%	<0.1	<8
Sodium chloride (NaCl)	g/kg	99.6	>97.0
Magnesium (Mg)	g/kg	0.05	<0.70
Potassium (K)	g/kg	0.07	<0.30
Calcium (Ca)	g/kg	0.094	<0.40
Iron (Fe)	mg/kg	6	<20
Copper (Cu)	mg/kg	<0.5	<1
Phosphorus (P)	mg/kg	<0.5	nd ^1^
Zinc (Zn)	mg/kg	<0.5	<1
Manganese (Mn)	mg/kg	<0.01	nd ^1^
Iodine (I)	mg/100 g	0.1	>0.07

^1^ Data not available.

**Table 4:** Change in color values of processed olives. NS = not significant; * = significant. Significant differences are indicated by different letters (*P* < 0.05).

Parameters	Carolea cv. (PGI)	Carolea cv. (Iodized-PGI)	ANOVA	Leucocarpa cv. (PGI)	Leucocarpa cv. (Iodized-PGI)	ANOVA
L	26.79 ± 16.40 ^a^	25.89 ± 13.87 ^a^	NS	65.09 ± 3.25 ^a^	62.45 ± 4.97 ^b^	*
a	16.07 ± 6.33 ^a^	15.98 ± 5.08 ^a^	NS	11.23 ± 1.75 ^a^	11.56 ± 2.01 ^b^	*
b	24.39 ± 13.67 ^a^	20.05 ± 11.04 ^b^	*	39.89 ± 3.00 ^a^	37.91± 3.93 ^b^	*
C	31.39 ± 9.55 ^a^	27.28 ± 7.66 ^b^	*	41.48 ± 3.00 ^a^	39.70 ± 3.80 ^b^	*

**Table 5:** Evaluation of sensory attributes. NS = not significant.

	Median	DSr	CVr %	CI Upper	CI Lower	Median	DSr	CVr %	CI Upper	CI Lower	
	**Carolea natural olives (PGI sea salt)**					**Carolea natural olives (iodized PGI sea salt)**					**ANOVA**
Salty	4.80	0.84	17.56	6.45	3.15	5.15	0.63	12.24	6.39	3.91	NS
Bitter	3.75	0.65	17.46	5.03	2.47	4.35	0.76	17.50	5.84	2.86	NS
Acid	7.00	0.55	7.83	8.07	5.93	7.00	0.44	6.31	7.87	6.13	NS
Hardness	3.60	0.71	19.78	5.00	2.20	3.95	0.77	19.48	5.46	2.44	NS
Fibrousness	4.60	0.39	8.54	5.37	3.83	4.10	0.34	8.38	4.77	3.43	NS
Crunchiness	4.05	0.58	14.35	5.19	2.91	4.15	0.70	16.76	5.51	2.79	NS
	**Leucocarpa natural olives (PGI sea salt)**					**Leucocarpa natural olives (iodized PGI sea salt)**					
Salty	4.00	0.29	7.37	4.58	3.42	4.15	0.40	9.66	4.94	3.36	NS
Bitter	6.05	0.36	5.95	6.76	5.34	6.75	0.47	7.03	7.68	5.82	NS
Acid	4.90	0.65	13.36	6.18	3.62	5.30	0.52	9.73	6.31	4.29	NS
Hardness	4.00	0.77	19.23	5.51	2.49	4.20	0.66	15.78	5.50	2.90	NS
Fibrousness	5.70	0.75	13.21	7.18	4.22	5.10	0.38	7.54	5.85	4.35	NS
Crunchiness	4.85	0.86	17.72	6.53	3.17	4.70	0.45	9.58	5.58	3.82	NS

**Table 6:** Microbial monitoring. Data are expressed in CFU/mL. * = significant. Significant differences are indicated by different letters (*P* < 0.05).

Samples	Carolea cv. (PGI)	Carolea cv. (Iodized-PGI)	ANOVA	Leucocarpa cv. (PGI)	Leucocarpa cv. (Iodized-PGI)	ANOVA
Total aerobic bacteria	-	-	-	-	-	-
LAB	-	-	-	-	-	-
Yeasts	3.5 × 10^5 a^	8.7 × 10^4 b^	*	3.1 × 10^2 a^	1.4 × 10^2 b^	*

**Table 7:** Tentative identification of the isolated yeasts. Identification percentages are shown between parentheses. ^1^ API 20 C AUX; ^2^ RapID Yeast Plus System.

Samples	Microorganisms
Carolea natural olives (PGI sea salt)	
1	*Candida krusei* [99.1] ^1^
2	*C. krusei* [99.1] ^1^
3	*C. krusei* [99.1] ^1^
4	*C. krusei* [99.1] ^1^
5	*C. krusei* [99.1] ^1^
Carolea natural olives (iodized PGI sea salt)	
1	*C. krusei* [99.1] ^1^
2	*C. krusei* [99.1] ^1^
3	*C. krusei* [99.1] ^1^
4	*C. krusei* [99.1] ^1^
5	*C. krusei* [99.1] ^1^
Leucocarpa natural olives (PGI sea salt)	
1	*Cryptococcus albidus* [95.0] ^2^
2	*Candida boidinii* [99.0] ^1^
3	*Candida famata* [99.0] ^2^
4	*C. boidinii* [99.0] ^1^
5	*Candida intermedia* [99.0] ^2^
Leucocarpa natural olives (iodized PGI sea salt)	
1	*C. boidinii* [99.0] ^1^
2	*C. intermedia* [99.0] ^2^
3	*C. krusei* [99.1] ^1^
4	*C. boidinii* [99.0] ^1^
5	*C. intermedia* [99.0] ^2^
